# Towards open, reliable, and transparent ecology and evolutionary biology

**DOI:** 10.1186/s12915-021-01006-3

**Published:** 2021-04-09

**Authors:** Rose E. O’Dea, Timothy H. Parker, Yung En Chee, Antica Culina, Szymon M. Drobniak, David H. Duncan, Fiona Fidler, Elliot Gould, Malika Ihle, Clint D. Kelly, Malgorzata Lagisz, Dominique G. Roche, Alfredo Sánchez-Tójar, David P. Wilkinson, Bonnie C. Wintle, Shinichi Nakagawa

**Affiliations:** 1 Evolution & Ecology Research Centre and School of Biological and Environmental Sciences, University of New South Wales, Sydney, NSW 2052 Australia; 2 Department of Biology, Whitman College, Walla Walla, WA 99362 USA; 3 School of Ecosystem and Forest Sciences, University of Melbourne, Burnley, Australia; 4 Department of Animal Ecology, Netherlands Institute of Ecology, NIOO-KNAW, 6708 PB Wageningen, Netherlands; 5 Institute of Environmental Sciences, Jagiellonian University, Kraków, Poland; 6 School of BioSciences, University of Melbourne, Parkville, Australia; 7 School of Historical and Philosophical Studies, University of Melbourne, Parkville, Australia; 8 Institute of Cognitive and Evolutionary Anthropology, University of Oxford, 64 Banbury Road, Oxford, OX2 6PN UK; 9 Département des Sciences Biologiques, Université du Québec à Montréal, Montreal, Canada; 10 Institute for Environmental and Interdisciplinary Science and Department of Biology, Carleton University, Ottawa, Ontario Canada; 11 Department of Evolutionary Biology, Bielefeld University, 33615 Bielefeld, Germany

## Abstract

Unreliable research programmes waste funds, time, and even the lives of the organisms we seek to help and understand. Reducing this waste and increasing the value of scientific evidence require changing the actions of both individual researchers and the institutions they depend on for employment and promotion. While ecologists and evolutionary biologists have somewhat improved research transparency over the past decade (e.g. more data sharing), major obstacles remain. In this commentary, we lift our gaze to the horizon to imagine how researchers and institutions can clear the path towards more credible and effective research programmes.

## Increasing transparency

Opaque research practices make it impossible to evaluate whether evidence generated by research is reliable, thereby stifling scientific progress [1]. As authors, peer reviewers, and readers of scientific papers, we simultaneously perpetuate, and are frustrated by, the information gap between producers and consumers of scientific studies. In the role of producers, we struggle to fastidiously document the long lifespan of research projects and are vulnerable to self-deception (e.g. rationalising statistical analyses that produce the most compelling results) [2]. In the role of consumers, too often we struggle to understand published articles, are left speculating about how and why particular conclusions were reached, and cannot build upon prior work. To help authors become reliable narrators of their own conduct and help readers better understand the published literature, proponents of reliable research have urged authors to document and report their research more transparently [3].

The lowest bar of academic reform is transparent reporting; with few exceptions (e.g. precise locations of endangered species), we can transparently share our research and ask the same of our community. In the past decade, most major ecology and evolutionary biology journals successfully mandated data sharing, with many journals signing onto the Transparency and Openness Promotion Guideline [3]. Researchers are also extending transparency efforts beyond traditional journal formats. Preprint servers (e.g. EcoEvoRxiv and bioRxiv) provide a history of in-preparation manuscripts, and authors can use online repositories (e.g. Open Science Framework) to document their research throughout the lifespan of their projects [1]. Having more of the research process made public would allow more people to learn from the mistakes and successes of others.

Changes in journals’ and funders’ policies can rapidly change researchers’ practices. Once leading journals in ecology and evolutionary biology started requiring open data upon publication, researchers complied, and more journals followed. Many journal guidelines now encourage authors to share their computer code too [4]. While the usability and quality of shared materials can be considerably improved—for example, by sharing pre-processed data with complete descriptions, and fully annotated code—gradually, more papers are becoming computationally reproducible (meaning their results can be reproduced from open data and code). Community expectations for shared materials in ecology and evolutionary biology should continue to rise, with some journals (e.g. *Ecology Letters*, *The American Naturalist*) poised to enlist ‘data editors’ during peer review.

## Preregistrations and registered reports

Transparent reporting requires transparent recording, because our current selves are often unreliable narrators of our past conduct. For example, perhaps our past-self tried multiple types of analyses before focusing on the clearest result, but our current-self, returning to these results months later, only remembers the statistically significant findings (‘*p*-hacking’ and ‘cherry picking’) [5]. Blinded by hindsight, our study could seemingly test a hypothesis that we had not planned to test (HARKing = ‘Hypothesizing After Results are Known’). These and other common biases [2] result in information gaps between our current and former selves. Complete information is then inaccessible to research consumers, making it harder to assess research reliability.

Transparent recording begins by describing a planned study prior to key events (e.g. data collection, exploration, and modelling) in an unalterable and publicly available document [1]. These ‘preregistration’ documents can be made for any type of study and reduce the potential for researcher self-deception while revealing the breadth of primary research before the filter of publication [6]. For example, mandatory registries for planned clinical trials revealed publication bias in drug development research [7]. Beyond controlled experiments, preregistration templates are expanding to include exploratory, descriptive, and theoretical work (e.g. guidance for preregistering modelling studies: https://osf.io/2qbkc/).

The benefits of preregistrations are amplified by ‘registered reports’, a style of publication first trialled in 2013 (a list of participating journals, such as *BMC Biology* and *Conservation Biology*, can be found at https://cos.io/rr). Registered reports are accepted on the strength of their study rationale, methods, and planned analyses, freeing researchers from results anxiety. Whereas traditional journal articles are reviewed (and revised) after a study is completed, registered reports are reviewed before and after the results are known (allowing studies to be critiqued and improved before it is too late to fix major flaws). While the delay in data collection might be difficult for researchers expected to generate results quickly (e.g. in many countries, doctoral students are expected to write three or more publishable chapters within 3–6 years), overcoming cultural and institutional barriers to registered reports could drastically reduce publication bias, while improving research quality, at the level of both researchers and publishers.

## Replications

To build upon published research, we need to understand the conditions under which findings are expected to replicate (i.e. understand ‘context dependence’). Yet, despite multiple papers urging ecologists and evolutionary biologists to conduct more replications, and researchers generally agreeing that replications are worthwhile, ecology and evolutionary biology journals publish close to zero (< 0.05%) studies considered close replications (i.e. adhering as closely as possible to the methods of an original study) [8]. There is therefore a disconnect between researcher’s beliefs towards replications and their behaviours.

**Fig. 1:**
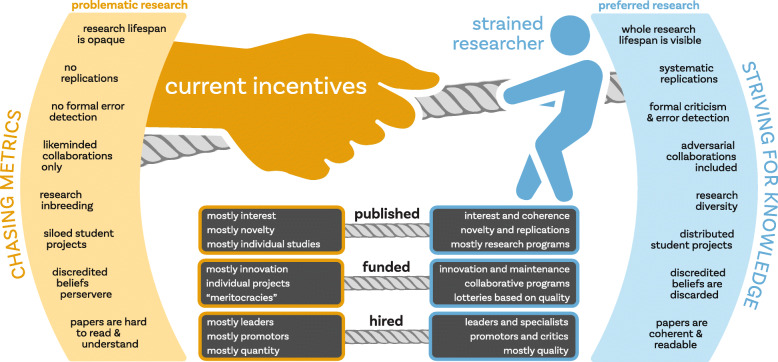
. The strained researcher is tugged away from their ideals by the incentives of the institutions they rely upon for employment and promotion. Practices and behaviours on the left-hand side of the tug-of-war (shaded orange) depict problems of the status quo, where research is focussed more on publishing papers than answering questions. Preferred practices and behaviours on the right-hand side of the tug-of-war (shaded blue) depict a vision for efficient and collaborative science aimed at credibly answering questions. To shift research practices towards reliability, three types of institutional incentives could change, as shown by grey boxes underneath the tug-of-war. First, journals and funders could quickly encourage validation of original research by publishing and funding replication studies. Less likely, journals could publish fewer, more comprehensive and coherent research programmes (both long-term studies and collections of smaller studies on the same research topic), thereby relieving pressures to oversell the importance of small studies. Second, employers could hire individuals with specialised expertise (e.g. data stewards, empiricists, statisticians, and writers), whose employment does not depend on particular research outcomes. Reducing the pyramid structure of academic career paths might promote a more diverse workforce that—without the pressure to maintain professional brands—could be quicker to discard discredited beliefs. Third, funding agencies could curb the benefits of self-promotion and irreproducible results by funding diverse teams, science maintenance (e.g. validation and error detection) as much as innovation, and by selecting randomly from projects that pass particular thresholds (i.e. grant lotteries). Grant lotteries are already being trialled by multiple funding agencies (e.g. the Fetzer Franklin Fund, the Health Research Council of New Zealand, and the Swiss National Science Foundation), but their effects on the reliability of research will depend on which metrics are used to select entrants into the lottery

Aligning the beliefs and behaviours of researchers requires a change in incentives (Fig. 1) [9]. Many researchers have suggested interventions to promote replication studies, including incorporating replications into trainee programmes (e.g. thesis chapters), dedicated funding and journal sections for replications, and requiring replications of short-term studies prior to publication. It would be easier to design close replication studies—and their results would be easier to interpret—if authors of primary studies specified when and where they would expect their findings to generalise (e.g. ‘Constraints on Generality’ statements). For studies that are logistically infeasible to closely replicate (e.g. isolated populations), attention can still be given to computational reproducibility and the robustness of results to alternative analysis decisions. Studies grounded in clearly defined theories can also be subject to conceptual replications, where the same hypotheses are tested in different biological contexts [8].

## Transparency is necessary but not sufficient

**Fig. 2:**
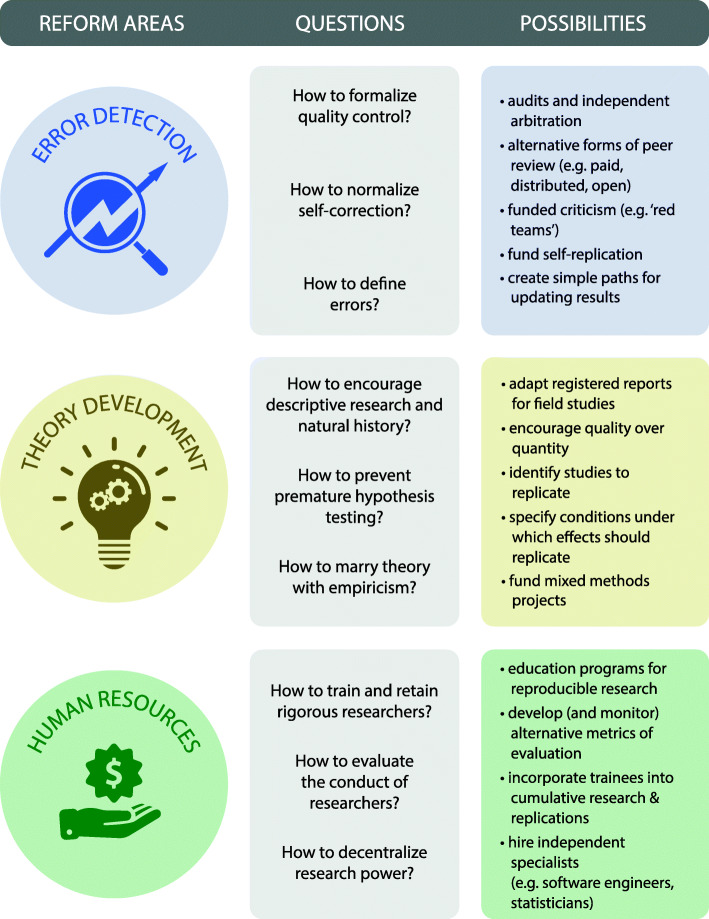
Three areas for reform to relieve research strain, outstanding questions for meta-research, and possible answers. **Error detection:** researchers need to be able to distinguish between reliable and unreliable research. A better system of quality control (both prior to and post-publication) might discourage research practices that inflate the rate of false-positive findings in the research literature (e.g. selective reporting; *p*-hacking; HARKing). At the same time, there should be incentives for researchers to remedy mistakes in their previous work, for example, through ‘living’ papers that can be easily updated. A more drastic change would be to require self-contained studies to be replicated, and for published results from long-term field studies to be revisited in subsequent years (e.g. before funding is renewed). **Theory development:** research in ecology and evolutionary biology sometimes fails to traverse the space between speculation and theory. In addition to hypothesis testing, answering big questions requires space for descriptive and exploratory research [10]. Detailed descriptions of natural history help calibrate theoretical models, and predictions of models should be tested in natural settings. To specify conditions under which findings are expected to replicate, authors can include ‘constraints on generality’ statements alongside inferences. When un-expected results are attributed to ‘context dependence’, specific contexts can be tested with new data. For cumulative research, foundational studies can be validated with close replications, and their generality assessed in different settings. **Human resources:** education programmes could increase the ability of researchers to work transparently and reproducibly, but honing these skills and conducting rigorous research is too often unrewarded. Any change to evaluation metrics requires careful consideration and measurement of unintended consequences (e.g. how to ensure costs are not disproportionately borne by less well-resourced research groups and universities). Much published research represents independent projects conducted by trainees, but reliability might be increased by coordinating multiple trainees on the same projects (including replication projects) and providing secure employment to people with specialised expertise (who can be professionally indifferent to the outcome of a particular study)

The information afforded by greater transparency only helps us discriminate between studies if we care to look (Fig. 2). Transparency alone does not prevent errors, nor does it guarantee that research helps to build and test strong theories. For example, methods might not measure what the authors claim to be measuring, and authors might not specify their claims precisely enough to be falsifiable. If preregistrations and supplementary materials are not read, data are not examined, analyses are not reproduced, and, crucially, close replications are not conducted or published, then our mistakes will not be identified. Researchers will always make mistakes, but changed incentives could encourage errors to be corrected and dissuade researchers from rushing into hypothesis testing, cutting corners, or fabricating results [9]. Common wisdom within the scientific community is that fraud is so rare as to be ignorable, but we cannot really know, as we do not really check; mechanisms to detect, investigate, and prosecute cases of fraud and research misconduct are under-resourced and not standardised across institutions. The dearth of formalised error detection in ecology and evolutionary biology suggests that we do not live up to the scientific ideal of organised scepticism.

## Changing incentives to relieve researcher strain

Many current institutional incentives foster irreproducible research. Major employers and funding agencies generally reward researchers for high publication outputs that attract a lot of attention, often without much regard for their reliability, selecting for productivity and hyperbole at the expense of rigour. Researchers in insecure employment might feel compelled to exaggerate the importance of their work to accrue more citations, and hastily publish papers that contribute little, if anything, to addressing specific research questions. Those who feel stifled by these publication pressures often simply leave academia, reducing the diversity of perspectives amongst people who stay [9]. Even those with secure employment are not spared from researcher strain. Tenured researchers can feel invested in their academic offspring, beholden to the expectations of their institution, or simply driven to match the outputs of their peers.

The problems described above sound bleak, but incentives are not immutable. Reform is possible and has already begun. For example, there are international efforts to change the way researchers are evaluated (e.g. the San Francisco Declaration on Research Assessment – DORA, and the Hong Kong Principles), which would relieve some of the strain on researchers (Fig. 1). Critics of reform might argue that there are inevitable inefficiencies in a complex system, science has always been a flawed human endeavour, and too many regulations risk stifling creativity. But while negative consequences of any regulations should be carefully monitored with meta-research, there is ample evidence that academic research could often be better than it currently is. Rather than being mere naysayers, advocates for reform are optimistically working towards a better research landscape.

## A community society for change

Researchers can make progress along the road to more credible research by uniting to educate our communities in more transparent and reliable research practices, and advocating for these practices to be valued by journals and funders. All of us (the authors) are founding members of the newly formed Society for Open, Reliable, and Transparent Ecology and Evolutionary biology (SORTEE: http://sortee.org, and @SORTEcoEvo on Twitter). SORTEE will absorb the previous efforts of the Tools for Transparency in Ecology and Evolutionary biology (https://osf.io/g65cb/) and is inspired by other researcher-driven organisations, such as the Center for Open Science, Society for the Improvement of Psychological Science, and the UK Reproducibility Network. As well as promoting transparent research practices, SORTEE aspires to foster communities of researchers who are passionate about improving research and institutional incentives in ecology and evolutionary biology.

At the time of writing, the COVID-19 pandemic has strained the university sector and funding agencies, but many researchers—caught between the accelerating demands of their profession and a desire to generate reliable results—were already feeling strained. This strain hurts individuals and threatens trust in science, especially on topics that are politically charged, and the disconnect between our ideals and actions could be worsened by the fiscal worries of our institutions. We can attempt to relieve this strain by reconsidering the type of research we want to be doing with scarce resources and by advocating for institutional change. Let us emerge from this period of uncertainty with renewed determination to conduct, and be valued for, open, reliable, and transparent research.

## Data Availability

No data or materials were presented in this manuscript.
